# The Expression of Genes *CYP1A1*, *CYP1B1*, and *CYP2J3* in Distinct Regions of the Heart and Its Possible Contribution to the Development of Hypertension

**DOI:** 10.3390/biomedicines12102374

**Published:** 2024-10-17

**Authors:** Maria L. Perepechaeva, Natalia A. Stefanova, Alevtina Y. Grishanova, Nataliya G. Kolosova

**Affiliations:** 1 Institute of Molecular Biology and Biophysics, Federal Research Center for Fundamental and Translational Medicine, Timakova Str. 2, Novosibirsk 630060, Russia; agrish@niimbb.ru; 2 Institute of Cytology and Genetics, Siberian Branch of Russian Academy of Sciences, 10 Akad. Lavrentiev Ave., Novosibirsk 630090, Russia; stefanovan@bionet.nsc.ru (N.A.S.); kolosova@bionet.nsc.ru (N.G.K.)

**Keywords:** hypertension, OXYS rats, heart chamber, CYP1B1, CYP2J3, CYP1A1

## Abstract

Background: It is believed that alterations in the functioning of the cytochrome P450 (CYP), which participates in metabolic transformations of endogenous polyunsaturated fatty acids (PUFAs) (with the formation of cardioprotective or cardiotoxic products), affects the development of age-related cardiovascular diseases and reduces the effectiveness of some cardioselective drugs. For example, CYP2J2 activation or CYP1B1 inhibition protects against the cardiovascular toxicity of anticancer drugs. It is currently unclear whether CYPs capable of metabolizing arachidonic acid and ω-3 PUFAs to vasodilatory and vasoconstrictive derivatives are expressed in all heart regions. Methods: The work was performed on senescence-accelerated OXYS rats featuring elevated blood pressure, OXYSb rats (an OXYS substrain with normal blood pressure), and Wistar rats as a “healthy” control. The mRNA level was determined in the right and left ventricles, the right and left atria, and the aorta of 1-, 3-, and 12-month-old rats. Results: We showed that all heart regions express CYPs capable of metabolizing arachidonic acid and ω-3 PUFAs and revealed significant differences between heart regions both in the mRNA level of genes *CYP1B1*, *CYP2J3*, and *CYP1A1* and in the time course of expression changes with age. Conclusions: We noticed that expression levels of these CYPs in the heart regions and aorta differ between hypertensive OXYS rats, normotensive OXYSb rats, and healthy Wistar rats but could not detect any clear-cut patterns associated with the hypertensive status of OXYS rats.

## 1. Introduction

Cardiovascular diseases remain the leading cause of death in the world, and their prevalence increases with age [1,2,3,4]. The most widespread among them is hypertension, which afflicts every third adult person [5]. The development of hypertension is mediated by several factors, including disturbances of the renin–angiotensin system, enhanced sympathetic activity, and inflammatory processes; these factors lead to excessive vasoconstriction, impaired sodium excretion, the enlargement of extracellular-fluid volume, and an increase in cardiac output [2,4,5].

Eicosanoids, i.e., metabolic products of arachidonic acid [one of the most common polyunsaturated fatty acids (PUFAs) in the human body] [6,7,8], and some metabolites of ω-3 PUFAs [9,10] take part in the pathogenesis of cardiovascular diseases, including hypertension.

Eicosanoids are synthesized within the human body in three known ways: via the cyclooxygenase pathway, through the lipoxygenase pathway, and via cytochromes of the P450 family. The participation of cyclooxygenase and lipoxygenase pathways’ products in pathological processes has been investigated well [11,12,13,14]. The eicosanoids formed with the help of P450 cytochromes are epoxy, hydroxy, and dihydroxy derivatives [7,11,15]: epoxyeicosatrienoic (EET) and hydroxyeicosatetraenoic (HETE) acids and some of their derivatives [6,11,16]. EET and HETE acids have a wide range of biological effects and function as secondary messengers of hormones, of growth factors, and of cytokines that regulate cardiovascular and several other physiological processes [17,18].

An imbalance in the biosynthesis of eicosanoids (products of CYP enzymes) is associated with the development of such pathological processes as hypertension and myocardial hypertrophy [17,18]. The role of EET and HETE acids in the pathogenesis of hypertension has been demonstrated both in animal experiments and in studies on enzyme polymorphisms in humans [19,20].

In most mammalian tissues, the main arachidonic-acid epoxygenases are CYP2C and CYP2J and to some extent CYP1A2 and CYP1B [6,11,21,22]. EET acids have positive effects on functions of the cardiovascular system: vasodilatory, antihypertensive, cardioprotective, renoprotective, antiatherosclerotic, and anti-inflammatory [6,21]. EET acids support kidney function by influencing the arteriolar tone and workings of epithelial sodium channels and by changing sodium absorption in proximal tubules and cortical collecting ducts [19,23].

Arachidonic-acid hydroxylation reactions predominantly involve CYP4A and CYP4F but also CYP2E and CYP2J and to some extent CYP1A2 and CYP1B [17,18,20,22]. The proinflammatory and vasoconstrictive effects of 20-HETE acid, which is mainly a product of metabolism carried out by CYP4A and CYP4F—and much less by CYP1A1 and CYP1B1—have been characterized well [22]. CYP1A1 and CYP1B1 are expressed in vascular endothelial cells, cardiomyocytes, and cardiac fibroblasts [24]. They are both under the transcriptional control of aryl hydrocarbon receptor (AhR) but play opposite roles in hypertension pathogenesis [24].

Several ω-3 PUFA metabolites that have potent vasodilatory properties are metabolized by P450 cytochromes [9,10]. For example, cytochrome P4501A1 (CYP1A1) metabolizes n-3 PUFAs to vasodilatory products [25].

The mechanisms underlying the development of hypertension may be linked with expression profiles of CYP enzymes [26]. CYP1B1 is known to mediate the angiotensin-II-induced activation of NADPH oxidase, the formation of reactive oxygen species, and the migration, proliferation, and hypertrophy of vascular smooth muscle cells [27]. Experiments on rodents have shown that CYP1B1 contributes to the development of (i) the hypertension caused by angiotensin II administration and (ii) the associated pathophysiological changes: cardiac hypertrophy, an enhanced vascular response to vasoconstrictors, the increased production of reactive oxygen species, and endothelial dysfunction [28,29]. Additionally, the reactive oxygen species formed under the action of CYP1B1 stimulate sympathetic activity [29]. After the administration of an CYP1B1 inhibitor or after a knockout of the *Cyp1b1* gene, these pathological changes are minimal [28].

Thus, the CYP enzymes producing vasoconstrictive and vasodilatory eicosanoids may take part in the pathogenesis of age-related cardiovascular diseases and are potential diagnostic markers and/or therapeutic targets in hypertension.

Several antihypertensive drugs, including angiotensin-converting enzyme inhibitors (captopril), β-blockers (alprenolol, carvedilol, metoprolol, and propranolol), and calcium channel blockers (diltiazem, felodipine, nimodipine nifedipine, and verapamil) are metabolized by different CYP enzymes, and accordingly large individual differences in treatment responses have been registered [30]. The major CYP enzymes that metabolize antihypertensive drugs are CYP3A4, CYP2D6, CYP1A1, CYP1A2, CYP2E1, and CYP2C9 [30].

It is known that inhibitors of CYP1B1 (which produces cardiotoxic metabolites), e.g., resveratrol (known for its antioxidant and cardioprotective effects) can be therapeutic agents for hypertension [31]. Theoretically, such agents may be activators of CYP2J3 (which produces cardioprotective metabolites) because gene therapy based on *CYP2J3* is reported to lower blood pressure in rat models of fructose-induced hypertension [32].

A quarter of a century ago, [33] howed that the expression of P450 monooxygenases’ genes varies significantly among heart regions, and, in their opinion, these differences may be implicated in the insufficient effectiveness of some cardioselective drugs. Nonetheless, their research has not progressed further, and, today, it is not clear whether CYP enzymes capable of metabolizing arachidonic acid and ω-3 PUFAs to their vasodilatory and vasoconstrictive derivatives are expressed in all chambers of the heart. It has been experimentally demonstrated that products of arachidonic-acid metabolism—EET and HETE acids—with the participation of CYP enzymes actively take part in the pathogenesis of cardiovascular diseases [34,35]. Therefore, determining the localization and expression of the CYP enzymes associated with eicosanoids’ metabolism in the cardiovascular system is important for understanding the pathological role of these enzymes. The aim of the present work was to quantify the mRNA expression of genes *CYP1A1*, *CYP1B1*, and *CYP2J3* in different regions of the heart and in the aorta and to assess a possible contribution of expression changes to the development of hypertension. The study was performed on senescence-accelerated OXYS rats, which, simultaneously with moderately elevated blood pressure, develop an early phenotype similar to geriatric diseases in humans, including hypertrophic cardiomyopathy, cataract, retinopathy similar to age-related macular degeneration in humans, and signs of Alzheimer’s disease [36,37,38,39]. The main differences between Wistar and OXYS rats emerge at the age of 3 months, i.e., during the active manifestation of signs of accelerated senescence in OXYS rats. At this age, according to ECG data, in the myocardium of OXYS rats, there are already small functional changes, which progress rapidly with age. As a consequence, at age 12 months, histopathological examination reveals typical signs of cardiomyopathy, such as myocardial hypertrophy, interstitial edema around coronary vessels, and myocardial fibrosis [36]. In the present study, healthy Wistar rats and rats of substrain OXYSb served as controls. The mRNA level was determined in right and left ventricles, right and left atria, and in the aorta in the period preceding the development (age 1 month), during small functional changes (3 months), and at the well-pronounced stages (12 months) of the typical signs of cardiomyopathy in OXYS rats. Healthy Wistar rats and rats of substrain OXYSb served as controls. OXYSb rats were derived from original OXYS rats of the 58th generation and differ from the parental strain by normal blood pressure and the delayed development of signs of aging [40].

## 2. Materials and Methods

### 2.1. Animals

The work was performed on male Wistar, OXYS, and OXYSb rats at the multi-access center Genetic Resources of Laboratory Animals at the Institute of Cytology and Genetics, the Siberian Branch of the Russian Academy of Sciences (ICG SB RAS). The animals were kept under a 12 h light/12 h dark regimen and received a standard laboratory diet and water ad libitum. Rats aged 1 to 12 months were used in the experiments.

The strain of prematurely aging (also hypertensive) rats—OXYS—was obtained as described previously [41]. In the 58th generation, the OXYSb substrain spontaneously emerged from the main strain (OXYS) as normotensive and also featured later development (and a mild course) of retinopathy [40]. The research was performed on animals of the 110th generation.

Arterial blood pressure in rats was measured under weak ethyl ether anesthesia (to prevent psychomotor sensitization) by the indirect sphygmographic method [42] in the tail at the age of 2 months in 25 animals of each strain (i.e., each genotype). To minimize the influence of anesthesia itself on blood pressure levels, all measurements of blood pressure in rats were carried out in identical settings.

At ages 1, 3, and 12 months, rats were anesthetized with CO_2_ and decapitated. Heart chambers and aortic sections were separated and frozen in liquid nitrogen until use.

Animal breeding, maintenance, and experimental procedures were carried out in accordance with the Directive 2010/63/EU of the European Parliament and of the European Council of 22 September 2010 and was approved by the Commission on Bioethics at the ICG SB RAS (decision # 34 of 15 June 2016), Novosibirsk, Russia. Every effort was made to minimize the number of animals used and their discomfort.

### 2.2. RNA Isolation and RT-PCR

RNA was extracted from tissue samples using the TRI-Reagent Kit (Ambion Inc., Austin, TX, USA) according to the manufacturer’s instructions and was treated with DNase (Promega Corporation, Fitchburg, WI, USA) also according to the manufacturer’s instructions. The RNA pellets were dissolved in 1 mM sodium citrate (pH 6.5) containing 1× RNASecure Reagent (Ambion Inc., Austin, TX, USA). RNA samples were stored at −20 °C until analysis.

RNA was converted to cDNA via reverse transcription (RT) by means of M-MuLV reverse transcriptase (Promega) according to the manufacturer’s instructions.

mRNA of the studied genes was quantified by real-time PCR according to the TaqMan principle on an iCycler CFX96 real-time PCR detection system (Bio-Rad Laboratories, Hercules, CA, USA). *GAPDH* served as a normalization control (housekeeping gene).

Primer sequences were selected in the Primer3Plus software and were as follows: *CYP1B1* [forward 5′-GGCATCGCACTTGTACTTCG-3′, reverse 5′-CACCAGAGCCTGATGGATGG-3′, probe 5′(FAM)-TCTCGCCATTCAGCACCACCACGG-(BHQ1)3′]; *CYP2J3* [forward 5′-CAATTCAGAATGTCCGTCACCAT-3′, reverse 5′-TCTTCGACATCACAGCCTAGGAA-3′, probe 5′(FAM)-CCCCAGTCAGTCACCGCCTTTGC-(BHQ1)3′]; *CYP1A1* [forward 5′-CCAAACGAGTTCCGGCCT-3′, reverse 5′-TGCCCAAACCAAAGAGAATGA-3′, probe 5′(FAM)-TTCTCACTCAGGTGTTTGTCCAGAGTGCC-(BHQ1)3′]; and *GAPDH* [forward 5′-CAAGGTCATCCATGACAACTTTG-3′, reverse 5′-GGGCCATCCACAGTCTTCTG-3′; probe 5′(FAM)-ACCACAGTCCATGCCATCACTGCCA-(BHQ1)3′].

The reaction mixture was composed of qPCRmix-HS buffer (Evrogen, Moscow, Russia) or BioMaster HS-qPCR (Biolabmix, Novosibirsk, Russia), a primer mix (consisting of the probe at a final concentration of 100 nM and forward and reverse primers at a final concentration of 200 nM each), and 500 ng of cDNA. The reaction was carried out under the following conditions: heating at 95 °C for 3 min and then 40 cycles of denaturation at 95 °C for 15 s and annealing/elongation at 60 °C for 30 s. cDNA samples were analyzed as three technical replicates in two independent experiments. For each experiment, calibration curves were constructed using serial dilutions (from 1:3 to 1:27) of cDNA. A mixture of aliquots of all cDNA samples served as standard cDNA.

### 2.3. Data Analysis

The raw data obtained from PCR were processed by the calibration graph method in the software that came with the iCycler CFX96. Statistical analysis was performed in the STATISTICA 13.0 (StatSoft Inc., Tulsa, OK, USA). To evaluate the significance of differences between groups of samples, one-way ANOVA was employed followed by post hoc analysis by the Newman–Keuls method. Data are presented as mean ± SEM.

## 3. Results

### 3.1. Blood Pressure in OXYS, OXYSb, and Wistar Rats

Blood pressure was measured in OXYS, OXYSb, and Wistar rats at the age of 3 months. In agreement with previously obtained findings, the highest blood pressure was noted in OXYS rats: 154.1 ± 2.5 mm Hg, which was statistically significantly higher than that in OXYSb rats (130.2 ± 4.1 mm Hg; *p* < 0.01) and in Wistar rats (127.1 ± 2.0 mm Hg; *p* < 0.01).

### 3.2. CYP mRNA Levels in the Heart Chambers and Aorta of OXYS, OXYSb, and Wistar Rats

The quantitation of *CYP1B1*, *CYP2J3*, and *CYP1A1* mRNAs in the heart (left and right atria and ventricles) and in the aorta of hypertensive rats (OXYS), normotensive rats (OXYSb), and “healthy” rats (Wistar) was conducted at ages 1, 3, and 12 months.

#### 3.2.1. Levels of CYP1B1 mRNA

As shown by one-way ANOVA, the level of *CYP1B1* mRNA (Table 1) in the heart and aorta depended on the genotype (F_2,308_ = 3.8, *p* = 0.022) and was lower in OXYS rats. By the age of 3 months, the expression of the *CYP1B1* gene increased, whereas by age 12 months, it decreased in rats of all three strains (F_2,308_ = 41.8, *p* < 0.0001). Furthermore, the lowest level of *CYP1B1* mRNA was found in the atria, and the highest was found in the aorta (F_2,308_ = 15.1, *p* < 0.0001).

In the atria, the amount of *CYP1B1* mRNA gradually went up with age in rats of all three strains (F_2,123_ = 18.3, *p* < 0.0001). It should be noted that the nature of gene expression differed between the right and left atria of rats. For instance, the level of *CYP1B1* mRNA was higher in the left atrium than in the right one in rats of all three strains at ages of 1 and 12 months (*p* < 0.05) but was lower only in Wistar rats at the age of 3 months (*p* < 0.05). As compared to Wistar rats, in OXYS rats at age 1 month, this gene’s expression was lower in the left atrium, whereas at age 3 months, it was lower in the right atrium (*p* < 0.05).

In the ventricles, the amount of *CYP1B1* mRNA gradually increased with age in rats of all three strains (F_2,130_ = 6.6, *p* < 0.002), but, in OXYS rats, this amount was lower (F_2,130_ = 3.1, *p* < 0.05). Significant differences in the magnitude of this gene’s expression between right and left ventricles were found only in OXYSb rats: the level of *CYP1B1* mRNA at the age of 1 month was lower in the left ventricle than in the right one and vice versa at age 12 months (*p* < 0.05).

In the aorta, the amount of *CYP1B1* mRNA depended on age (F_2,55_ = 19.7, *p* < 0.0001): by the age of 3 months, this parameter increased, but, by age 12 months, it diminished in rats of all three strains. In this context, the factors “genotype” and “age” interacted significantly (F_4,55_ = 4.4, *p* < 0.004). A comparison of group means indicated that as compared to Wistar rats, the level of *CYP1B1* mRNA in the aorta of OXYS rats was lower at the age of 3 months and higher at 12 months (*p* < 0.05).

#### 3.2.2. The Level of CYP2J3 mRNA

The data on *CYP2J3* mRNA quantification are presented in Table 2. The examination of the results showed that the level of *CYP2J3* mRNA in the heart chambers and aorta depended on the genotype (F_2,315_ = 5.7, *p* < 0.004). This parameter was lower in OXYS rats; it was the lowest in the atria and the highest in the aorta of rats of all three strains (F_2,315_ = 37.6, *p* < 0.0001).

In the atria, the amount of *CYP2J3* mRNA went up by the age of 3 months and was still at the same level at age 12 months in rats of all three strains (F_2,133_ = 30.8, *p* < 0.0001), but, in OXYS rats, this parameter was lower (F_2,133_ = 3.6, *p* < 0.03). In terms of this gene’s expression differences between the right and left atrium, significant differences were found in 1-month-old OXYSb rats and in 12-month-old rats of all three strains: the level of *CYP2J3* mRNA in the left atrium was higher (*p* < 0.05).

As revealed by a comparison of group means, in the cardiac ventricles, interstrain differences in the amount of *CYP2J3* mRNA were significant only at the age of 3 months: it was lower in OXYS rats than in Wistar and OXYSb rats (*p* < 0.05). It should be pointed out that there were differences in the time course of age-related changes in *CYP2J3* expression in the ventricles among the three strains. In Wistar rats, the level of *CYP2J3* mRNA in the right ventricle increased by the age of 3 months (*p* < 0.05) and was still at the same level at 12 months; in the left ventricle, it gradually went up with age. In OXYSb rats, in the right ventricle, this parameter declined starting from the age of 3 months (with marginal significance: *p* = 0.061), whereas in the left ventricle, it did not change with age. In OXYS rats, this gene’s expression in the right ventricle gradually diminished with age; in the left ventricle, it decreased by age 3 months (*p* < 0.05) and increased by age 12 months (*p* < 0.05).

In the aorta, the level of *CYP2J3* mRNA depended on the genotype (F_2,54_ = 3.4, *p* < 0.05) and was lower in OXYS and OXYSb rats than in Wistar rats. The amount of *CYP2J3* mRNA depended on age: in Wistar rats, it increased by age 3 months and diminished by age 12 months; on the contrary, in OXYS and OXYSb rats, it decreased by age 3 months and increased by 12 months of age.

#### 3.2.3. The Level of CYP1A1 mRNA

The data on *CYP1A1* mRNA quantitation are given in Table 3. The level of *CYP1A1* mRNA in various chambers of the heart was found to vary only slightly depending on the genotype. As the data analysis showed, the amount of *CYP1A1* mRNA in chambers of the heart and in the aorta rose by the age of 3 months and declined by age 12 months in rats of all three strains (F_2,303_ = 7.6, *p* < 0.001). The lowest expression of this gene was registered in the aorta, and the expression was the highest in cardiac ventricles (F_2,303_ = 8.1, *p* < 0.001).

In the atria of rats of all three strains, the level of *CYP1A1* mRNA increased by the age of 3 months and decreased by age 12 months to the level of 1-month-old animals (F_2,128_ = 67.4, *p* < 0.0001), while this gene’s expression was higher in the left atrium than in the right one at all ages.

In cardiac ventricles, the amount of *CYP1A1* mRNA was influenced by the age factor (F_2,121_ = 3.8, *p* < 0.03): in Wistar rats, this gene’s expression went up by the age of 12 months (*p* < 0.05), whereas in OXYS and OXYSb rats, it was up already by age 3 months (*p* < 0.05) and then stayed at this level. Additionally, in rats of all three strains, at ages 1 and 3 months, the level of *CYP1A1* mRNA was higher in the left ventricle than in the right one (*p* < 0.05), whereas at age 12 months, there were no differences between the ventricles. In this context, at the age of 12 months, the amount of *CYP1A1* mRNA was significantly higher in Wistar rats than in OXYS and OXYSb rats.

No significant changes with age or interstrain differences in *CYP1A1* expression were detectable in the aorta.

## 4. Discussion

In this study, in different regions of the heart, we for the first time assessed expression levels of the CYP enzymes that are capable of metabolizing arachidonic acid and ω-3 PUFAs to derivatives having vasodilatory and vasoconstrictive properties and uncovered significant differences. We also demonstrated that levels of *CYP1B1*, *CYP2J3*, and *CYP1A1* mRNAs in the right and left heart chambers or the aorta differ between hypertensive rats (OXYS strain), normotensive rats (OXYSb), and healthy rats (Wistar). It is worth mentioning that the average level of *CYP1B1* and *CYP2J3* mRNAs in the right and left chambers of the heart proved to be low in OXYS rats. Furthermore, amounts of *CYP1B1* and *CYP2J3* mRNAs in rats of all strains were found to be the lowest in the atria and the highest in the aorta, whereas the mRNA expression of the *CYP1A1* gene turned out to be the lowest in the aorta and the highest in cardiac ventricles.

It is noteworthy that the main differences between the rat strains emerge at the age of 3 months (i.e., during the active manifestation of signs of accelerated senescence in OXYS rats), and, at age 12 months, histopathological examination reveals typical signs of cardiomyopathy [36]. On the other hand, mRNA levels of the genes under study in various regions of the heart of 1-year-old OXYS rats—as well as 1-month-old ones—did not differ significantly from those in Wistar and OXYSb rats in the current work.

It should be mentioned that in the heart of OXYS rats, lower levels of both CYP1B1 and CYP2J3 mRNAs were observed, although the actions of products of these metabolic enzymes have different directions. Products of metabolism by CYP1B1 are cardiotoxic [41,43]. In the heart, CYP1B1 is responsible for the biosynthesis of medium-chain HETE acids: 5-, 8-, 9-, 11-, 12-, and 15-HETE [18,44]. For instance, 12-HETE acid is known to be a cardiotoxic compound [43], whereas a CYP1B1 inhibitor called resveratrol attenuates angiotensin-II-induced cellular hypertrophy by reducing CYP1B1 protein expression and the production of HETE acids in human RL-14 cardiomyocytes and rat H9c cardiomyoblasts [31]. The inhibition of CYP1B1 may protect against the cardiovascular toxicity of anticancer drugs by reducing the concentration of medium-chain HETE acids [45].

In spontaneously hypertensive rats (SHR strain), enhanced CYP1B1 activity is associated with left ventricular hypertrophy, whereas the inhibition of CYP1B1 attenuates the increase in blood pressure and limits such manifestations as elevated vascular reactivity, endothelial and renal dysfunction, and fibrosis [29,46] concurrently with a decrease in reactive oxygen species production and in NADPH oxidase activity and increased plasma concentrations of proinflammatory cytokines and catecholamines and enhanced activities of cardiac p38 MAPK, ERK, tyrosine kinase c-Src, and Akt [29]. On the contrary, EET acids (products of metabolism by CYP2J3) are known as cardioprotectors [43], which reduce blood pressure and act as vasodilators [23,47]. Furthermore, CYP2J2 and EET acids exert cardioprotective effects by modulating the cardiotoxicity of doxorubicin and possibly of other drugs [48].

Therefore, both cardioprotective CYP2J3 and cardiotoxic CYP1B1 are expressed at overall lower levels in cardiovascular tissues of hypertensive OXYS rats as compared to normotensive OXYSb rats and healthy Wistar rats. Nonetheless, the resulting biological effects seem to be cardiotoxic, possibly due to the participation of other, yet unknown factors.

CYP1A1 mostly catalyzes the formation of 16-19-HETE acids (anti-inflammatory and vasodilatory compounds), can make a small contribution to the biosynthesis of 20-HETE acid [43], and metabolizes n-3 PUFAs to vasodilatory products [25]. High blood pressure is observed in mice with a knockout of the *Cyp1a1* gene [25] or of the *AhR* gene; AhR is a regulator of *CYP1A* transcriptional activity [49]. Additionally, an allele of increased *CYP1A1* inducibility—CYP1A1*2A—seems to have protective properties toward peripheral circulation [50]. Our work revealed differences in the level of *CYP1A1* mRNA among different chambers of the heart, whereas in OXYS and OXYSb rats, this parameter increased by the age of 3 months in the atria and ventricles.

Changes in the expression of CYP enzymes with age may be connected with age-mediated epigenetic alterations, as demonstrated for *CYP2E1* [51], or with effects of oxidative stress, which has been shown to suppress *CYP1A1* expression in rat hepatocytes [52,53]. Oxidative stress is regarded as one of the key factors leading to the development of age-related diseases [54]. OXYS rats are more sensitive to oxidative stress than Wistar rats are [55], and this difference may be reflected in—among other things—expression levels of *CYP* genes. In turn, the activity of CYP enzymes—in particular, CYP1B1 and CYP1A1—itself is a factor enhancing oxidative stress [56]. As a consequence, feedback loops with various ultimate effects may arise.

It should be mentioned that only levels of CYP mRNAs were assayed in this work. Levels of CYP proteins and enzymatic activities may change disproportionately to the transcription level of the corresponding genes; therefore, it is difficult to interpret physiological significance of the changes—in levels of CYP mRNAs in various chambers of the heart and the aorta—observed in our study.

The yield of enzymes’ products and the activity of enzymes can be affected by mutations (e.g., single-nucleotide polymorphisms) in *CYP* genes, and these mutations in theory may underlie differences in genotypes between the rat strains being analyzed here. Such mutations can lead to the modification of protein structure and to alterations of the functional activity of enzymes. Additionally, different genotypes can feature dissimilar intensities of processes of CYP enzyme degradation. These characteristics may be a good subject for further research.

Consequently, we demonstrated that all chambers of the heart express the CYP enzymes capable of metabolizing arachidonic acid and ω-3 PUFAs to derivatives possessing vasodilatory and vasoconstrictive properties as well as revealed significant differences between the heart chambers both in the mRNA levels of *CYP1B1*, *CYP2J3*, and *CYP1A1* and in the time course of their changes with age. We also showed that expression levels of these CYP enzymes in the heart and aorta differ between hypertensive OXYS rats, normotensive OXYSb rats, and healthy Wistar rats, but we failed to detect any obvious patterns correlating with the hypertensive status of OXYS rats. It is possible that the hypertensive status of OXYS rats is determined by the vasoactive metabolites of arachidonic acid that are synthesized with the participation of other enzymes: cyclooxygenases or lipoxygenases.

## Figures and Tables

**Table 1:** Levels of CYP1B1 mRNA in the heart and aorta of Wistar, OXYS, and OXYSb rats.

Strain	Age, Month	Atrium			Ventricle			Aorta
		Right	Left	Mean ± SEM	Right	Left	Mean ± SEM	
Wistar	1	0.26 ± 0.04	0.99 ± 0.09 ^£^	0.65 ± 0.11	0.67 ± 0.17	0.63 ± 0.21	0.65 ± 0.13	0.74 ± 0.10
	3	1.33 ± 0.08 ^#^	0.76 ± 0.13 ^£^	1.02 ± 0.11 ^#^	1.18 ± 0.18	1.46 ± 0.13 ^#^	1.32 ± 0.11 ^#^	5.58 ± 1.36 ^#^
	12	0.54 ± 0.10 ^#^	1.75 ± 0.14 ^#£^	1.10 ± 0.19	0.66 ± 0.15 ^#^ *p* = 0.056	1.56 ± 0.62	1.08 ± 0.31	0.17 ± 0.17 ^#^
OXYS	1	0.31 ± 0.07	0.57 ± 0.07 *^£^	0.44 ± 0.06	0.46 ± 0.11	0.36 ± 0.06 ^+^	0.39 ± 0.05	0.91 ± 0.13
	3	0.82 ± 0.06 *^#^	0.90 ± 0.16	0.87 ± 0.10 ^#^	0.96 ± 0.22	0.77 ± 0.10 *^+#^	0.86 ± 0.12 *^#^	2.22 ± 0.83 *
	12	0.52 ± 0.12 ^#^	1.68 ± 0.18 ^#£^	1.14 ± 0.20	0.49 ± 0.12	2.51 ± 0.24 ^#^	1.42 ± 0.32 ^#^ *p* = 0.056 ^£^	0.92 ± 0.27 *
OXYSb	1	0.42 ± 0.06	0.71 ± 0.09 ^£^	0.56 ± 0.07	1.63 ± 0.39 *	0.81 ± 0.14 ^£^	1.16 ± 0.21 *	0.96 ± 0.19
	3	1.17 ± 0.16 ^#^	1.05 ± 0.11 ^#^	1.10 ± 0.09 ^#^	1.03 ± 0.18	1.18 ± 0.17	1.10 ± 0.12	2.21 ± 0.99
	12	0.75 ± 0.11	1.46 ± 0.22 ^£^	1.13 ± 0.16	0.75 ± 0.18	2.44 ± 0.46 ^#£^	1.60 ± 0.33	0.79 ± 0.44

Significant differences: * as compared with Wistar rats of the same age; ^+^ as compared with OXYSb rats of the same age; ^#^ as compared with the previous age in rats of the same strain; ^£^ as compared with a right-hand chamber of the heart in rats of the same strain. Data are presented as mean ± SEM (n = 3–8). The data are presented as an expression ratio of a target gene to a reference gene (*GAPDH*).

**Table 2:** Levels of *CYP2J3* mRNA in the heart and aorta of Wistar, OXYS, and OXYSb rats.

Strain	Age, Month	Atrium			Ventricle			Aorta
		Right	Left	Mean ± SEM	Right	Left	Mean ± SEM	
Wistar	1	0.57 ± 0.12	0.51 ± 0.06	0.54 ± 0.07	0.68 ± 0.14	0.88 ± 0.18	0.79 ± 0.12	1.93 ± 0.33
	3	1.30 ± 0.10 ^#^	1.25 ± 0.25 ^#^	1.27 ± 0.16 ^#^	1.19 ± 0.10 ^#^	0.98 ± 0.16	1.09 ± 0.09 ^#^ *p* = 0.056	3.01 ± 0.54
	12	0.79 ± 0.16 ^#^	1.55 ± 0.18 ^£^	1.17 ± 0.16	1.02 ± 0.20	1.74 ± 0.58	1.41 ± 0.33	1.38 ± 0.41 ^#^
OXYS	1	0.35 ± 0.05	0.44 ± 0.04 ^+^	0.39 ± 0.03 * *p* = 0.055	1.33 ± 0.35	0.92 ± 0.14	1.04 ± 0.14	1.83 ± 0.26
	3	0.89 ± 0.07 *^#^	0.88 ± 0.14 ^#^	0.89 ± 0.07 * *p* = 0.027 ^#^	0.82 ± 0.10 * ^+^ *p* = 0.051 ^#^ *p* = 0.064	0.51 ± 0.08 *^+#£^	0.66 ± 0.07 *^+#^	1.24 ± 0.30 *
	12	0.68 ± 0.13	1.35 ± 0.09 ^#£^	1.01 ± 0.12	0.54 ± 0.12	2.40 ± 0.37 ^#^	1.40 ± 0.32 ^#£^	1.65 ± 0.23
OXYSb	1	0.30 ± 0.05	0.69 ± 0.09 ^£^	0.50 ± 0.07	1.25 ± 0.28	1.10 ± 0.29	1.17 ± 0.20	1.98 ± 0.36
	3	1.07 ± 0.11 ^#^	0.96 ± 0.08 ^#^	1.02 ± 0.07 ^#^	1.10 ± 0.09	1.39 ± 0.17	1.23 ± 0.10	0.96 ± 0.22 *^#^
	12	0.52 ± 0.12 ^#^	1.45 ± 0.20 ^#£^	1.02 ± 0.18	0.75 ± 0.17 ^#^ *p* = 0.061	1.54 ± 0.31 ^£^	1.11 ± 0.20	1.34 ± 0.32

Significant differences: * as compared with Wistar rats of the same age; ^+^ as compared with OXYSb rats of the same age; ^#^ as compared with the previous age in rats of the same strain; ^£^ as compared with the right-hand chamber of the heart in rats of the same strain. Data are presented as mean ± SEM (n = 3–8). The data are presented as an expression ratio of a target gene to a reference gene (*GAPDH*).

**Table 3:** Levels of *CYP1A1* mRNA in the heart and aorta of Wistar, OXYS, and OXYSb rats.

Strain	Age, Month	Atrium			Ventricle			Aorta
		Right	Left	Mean ± SEM	Right	Left	Mean ± SEM	
Wistar	1	0.03 ± 0.01	0.18 ± 0.02 ^£^	0.11 ± 0.02	0.35 ± 0.09	0.11 ± 0.03 ^£^	0.21 ± 0.05	0.25 ± 0.12
	3	0.53 ± 0.16 ^#^	1.14 ± 0.24 ^#^ ^£^ *p* = 0.055	0.84 ± 0.16 ^#^	0.46 ± 0.07	0.11 ± 0.03 ^£^	0.27 ± 0.06	0.08 ± 0.03
	12	0 ^#^	0.19 ± 0.10 ^#^	0.10 ± 0.06 ^#^	0.21 ± 0.08 ^#^	0.51 ± 0.24 ^#^	0.33 ± 0.12	0.01 ± 0.01^#^ *p* = 0.053
OXYS	1	0.05 ± 0.01	0.14 ± 0.03 ^£^	0.09 ± 0.02	0.39 ± 0.19	0.10 ± 0.02 ^£^	0.18 ± 0.06	0.12 ± 0.05
	3	0.60 ± 0.16 ^#^	0.80 ± 0.12 ^#^	0.68 ± 0.11 ^#^	0.70 ± 0.06 * ^#^ *p* = 0.051	0.11 ± 0.02 ^£^	0.48 ± 0.08 *^#^	0.01 ± 0.01
	12	0 ^#^	0.03 ± 0.01 ^#£^	0.02 ± 0.01 ^#^	0.27 ± 0.08 ^#^	0.37 ± 0.10 ^#^	0.32 ± 0.06	0.37 ± 0.25
OXYSb	1	0.03 ± 0.01	0.14 ± 0.04 ^£^	0.08 ± 0.02	0.43 ± 0.16	0.09 ± 0.02 ^£^	0.22 ± 0.08	0.13 ± 0.05
	3	0.56 ± 0.10 ^#^	1.03 ± 0.11 ^#£^	0.76 ± 0.10 ^#^	1.05 ± 0.17 ^#^*	0.13 ± 0.02 ^£^	0.61 ± 0.14 *^#^	0.08 ± 0.04
	12	0 ^#^	0.14 ± 0.10 ^#^	0.07 ± 0.05 ^#^	0.42 ± 0.12 ^#^	0.20 ± 0.04	0.30 ± 0.07	0.10 ± 0.10

Significant differences: * as compared with Wistar rats of the same age; ^#^ as compared with previous age in rats of the same strain; ^£^ as compared with a right-hand chamber of the heart in rats of the same strain. Data are presented as mean ± SEM (n = 3–8). The data are presented as an expression ratio of a target gene to a reference gene (*GAPDH*).

## Data Availability

The raw data generated during and/or analyzed during the current study are available from the corresponding author upon reasonable request.
